# A universal tool for visualisation of networks and trees in Python

**DOI:** 10.12688/f1000research.173131.1

**Published:** 2025-12-09

**Authors:** Fabio Zanini

**Affiliations:** 1 School of Clinical Medicine, University of New South Wales, Sydney, New South Wales, 2052, Australia; 2 Ecology & Evolution Research Centre, University of New South Wales, Sydney, NSW, 2052, Australia; 3 UNSW Cellular Genomics Futures Institute, UNSW, NSW, 2052, Australia

**Keywords:** networks, graphs, trees, visualisation, phylogenetics, plotting, interactions.

## Abstract

**Background:**

Graphs or networks are ubiquitous data structures across scientific disciplines, from ecological and molecular to social networks. Visualisation is an important step to gain insight into network data, however most network analysis software packages, such as *igraph* and *NetworkX*, focus on statistical tools and offer limited visualisation options. Other packages offer powerful graphical user interfaces but rely on custom formats and implementations, which restricts interoperability with other scientific software.

**Methods:**

*iplotx* is a Python software package designed to act as a universal visualiser for any network analysis package. A simple yet rich declarative style grammar enables customisation of any visual element including edge geometry, marker size and shape, labels, and groupings. Rendering relies on custom Matplotlib artists, which enables broad interoperability with other data visualisation libraries.

**Results:**

*iplotx* supports eight different network and tree analysis packages (NetworkX, igraph, graph tool, Biopython, ETE, cogent3, dendropy, and scikit-bio) and implements a plug-in mechanism for universal input compatibility. It supports a wide spectrum of outputs including raster and vector images, animations, and interactive plots. The package implements unique features such as three-dimensional network visualisations and is fully compatible with the Python scientific software stack including seaborn, scipy, pandas, and Cartopy. Areas of application for *iplotx* include biomolecular and gene regulatory networks, protein-protein interactions, biochemical pathways, ecological networks, phylogenetics, and extend beyond biomedicine into economics, social, and transportation sciences.

**Conclusions:**

Compared to extant software, *iplotx* offers broader interoperability, richer styling, a large gallery of examples, and extensive code testing. This makes *iplotx* a robust choice for the visualisation of any networks and trees in biomedicine and beyond.

## Introduction

Graphs or networks are widely used to encode relationships between entities including people (social networks), species (e.g. food networks, phylogenies), places (e.g. transportation networks) and domain-specific objects (e.g. gene regulatory networks). Network analysis is an important research field across scientific disciplines and supported by a number of software packages including *NetworkX* ( Hagberg, Schult, and Swart 2008), *igraph* ( Antonov et al. 2023), and *graph-tool* ( Peixoto 2017) in Python and *rigraph* ( Antonov et al. 2023) and *statnet* ( Pavel N. Krivitsky, Mark S. Handcock and Hunter, David R. and Butts, Carter T. and Bojanowski, Michal and Klumb, Chad and Goodreau, Steven M. and Morris, Martina 2003/2024) in R. Among all networks, trees are often used to represent hierarchical relationships, such as animal ( Redelings and Holder 2017) and viral evolution ( Sagulenko, Puller, and Neher 2018; Drummond and Rambaut 2007). Mature software libraries that focus on or include manipulation and analysis of trees include *cogent3* ( Huttley et al. 2025), *dendropy* ( Moreno, Holder, and Sukumaran 2024), Environment for Tree Exploration ( *ETE*) ( Huerta-Cepas, Serra, and Bork 2016), *Biopython* ( Cock et al. 2009), and *scikit-bio* ( Team 2020).

The centrality of statistics notwithstanding, visualisation is an equally important tool to explore and understand a network’s topology and other properties. As mathematical objects, networks and trees exist outside of geometric spaces, therefore their visualisation requires two steps: i) Embedding the network in either a two- or a three-dimensional space, and ii) Visualising the resulting layout in a way that facilitates interpretation of the network properties. Network analysis libraries already implement many layout algorithms ( Antonov et al. 2023; Hagberg, Schult, and Swart 2008; Peixoto 2017).

Underscoring the importance of visualisation is the existence of domain-specific software that centers the entire workflow of network analysis and interpretation on a graphical user interface (GUI). Popular examples in this space include *Cytoscape*, developed for biomolecular networks ( Shannon et al. 2003), *SocNetV*, focused on social networks ( “Free and Open-Source Tool for Social Network Analysis,” n.d.), and *nextstrain*, dedicated to pathogen evolution ( Hadfield et al. 2018).

While visualisation functionality varies across these tools, they tend to fall into one of two categories. Some packages, including *NetworkX*, *igraph*, *cogent3*, and *dendropy* offer limited plot customisation and rely on standard plotting backends such as *Matplotlib* ( Hunter 2007), *plotly* ( Plotly Technologies Inc. 2015), and the terminal. Others, including *graph-tool (* *Peixoto 2017* *)*, *Cytoscape* ( Shannon et al. 2003), *SocNetV* (“Free and Open-Source Tool for Social Network Analysis,” n.d.) and *ETE* ( Huerta-Cepas, Serra, and Bork 2016), offer a higher level of customisation but use non-interactive or custom rendering backends, which precludes interoperability with other chart types beyond the network itself.

*iplotx* was developed to enable highly customisable network visualisation that is fully compatible with a generic plotting framework. By relying on a Matplotlib, it naturally supports animations, post-render editing, multiple output types (e.g. vector and raster files, interactive widgets), and compatibility with both Matplotlib features (e.g. other chart types, annotations, inset axes, colour bars) and a large library of third-party tools including seaborn ( Waskom et al. 2014) and scipy ( Virtanen et al. 2020). Unlike other tools, *iplotx* accepts data from multiple input libraries ( *NetworkX*, *igraph*, *graph-tool *, *Biopython*, *cogent3*, *ETE4*, *dendropy*, and *scikit-bio *) and includes a mechanism to extend compatibility to any input data stream. Because of this universal compatibility, *iplotx* guarantees identical visual appearance for networks and trees independent of how they were processed.

## Methods

### Implementation

*iplotx* follows best practices in free open-source software development. The codebase is versioned on GitHub has a stable API. Documentation ( https://iplotx.readthedocs.io) includes a reference library, a gallery of about 90 examples, and installation instructions. Code integrity is verified via continuous integration/development (CI/CD) pipelines and 232 unit tests covering >90% of the codebase. Code formatting tools and pre-commit hooks ensure high readability. User and contributor interactions take place online transparently and respectfully.

Technically, the library leverages custom Matplotlib artists ( Hunter 2007) to ensure a coherent appearance of nodes, edges, and other element types upon transformations (e.g. zoom, pan, different Density Per Inch (DPI) settings, animations). Two sets of coordinate transforms are used throughout the codebase: (i) Data units to define the location of the node centers and other artists’ properties, and (ii) Figure units to define node size, edge width, and similar “scale-free” properties. This dual coordinate system is consistent with the behaviour of general charting libraries which maintain marker size upon zoom.

### Operation

Installation is achieved via the standard PyPI repository: detailed instructions available on the website. The software package supports Python 3.10 or later and any platform covered by its dependencies, namely Matplotlib and pandas, which includes Linux, MacOS, and Windows. The package is imported as a Python module and used mainly via its *iplotx.network* and *iplotx.tree* functions. *iplotx* autodiscovers network analysis libraries at runtime and provisions input data providers based on the user’s specific installation. For example, if a user’s installation includes *igraph* but not *NetworkX*, *iplotx* will automatically activate the corresponding input data stream: no user input is required.

## Results

### Efficient, universal middleware between network analysis and rendering

**Figure 1.**
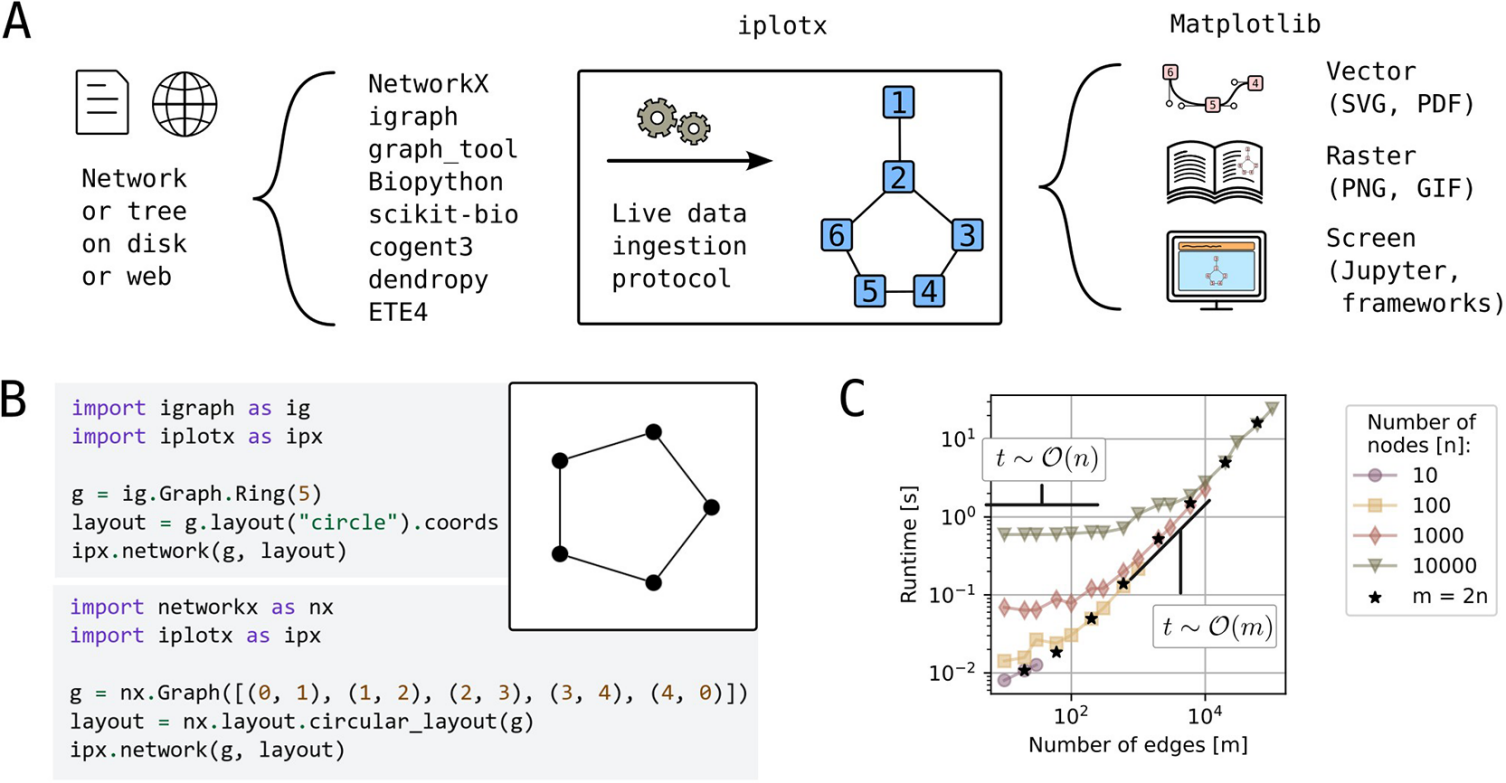
Overview of *iplotx’s* scope. **A)** Schematic representation of network visualisation workflows with *iplotx* as a universal intermediate stage between network/tree analysis libraries [REFs] and the Matplotlib rendering backend [REF]. **B)** Simple example of a network visualisation from two distinct libraries through *iplotx*, which guarantees identical appearance independent of the data provider. **C)** Runtime scalability for Erdős–Rényi graphs with varying number of nodes (n) and edges (m). Black stars indicate graphs with twice as many edges as nodes. All computations were performed on a consumer laptop (Dell Latitude 7440) and ran for less than a minute.

Network visualisation sits between network analysis and graphical rendering onto paper or screen. The goal of *iplotx* is to unify this intermediate stage for the Python ecosystem ( Figure 1A). For this purpose, *iplotx* was designed to accept input from multiple (currently eight) network and tree analysis libraries ( Antonov et al. 2023; Hagberg, Schult, and Swart 2008; McArthur et al. 2025; Team 2020; Huerta-Cepas, Serra, and Bork 2016; Cock et al. 2009). *iplotx* also implements simple network and tree data structures for users seeking to minimise external package dependencies. A separate mechanism enables external developers to add compatibility with *iplotx* to their own package. Internally, data from all input providers is represented in the same exact way, guaranteeing the same visual output independently of the input stream ( Figure 1B).

Consistent output on multiple rendering backends was achieved through tight integration with *Matplotlib* ( Hunter 2007), a popular library for data plotting that can export charts into multiple image formats (e.g. PNG, SVG) but also offers interactive renderers such as Tkinter, Qt, and GTK, which react to mouse and keyboard events and can be animated ( Figure 1A). *iplotx* objects can be combined with other artists such as bar, line, and scatter plots, images, and inset axes.

*N*etworks with hundreds of thousands of nodes and edges can be visualised within seconds on a consumer laptop ( Figure 1C). Empirical tests confirm the expected runtime complexity as O(n+m) where *n* and *m* are the number of nodes and edges: the backend needs to render vertices and edges individually. This level of efficiency is abundantly sufficient for many practical applications and can be further improved by strategic user choices (e.g. visualising a subset of or no edges). Examples of practical applications are given below.

### Simple yet expressive styling of networks and trees

**Figure 2.**
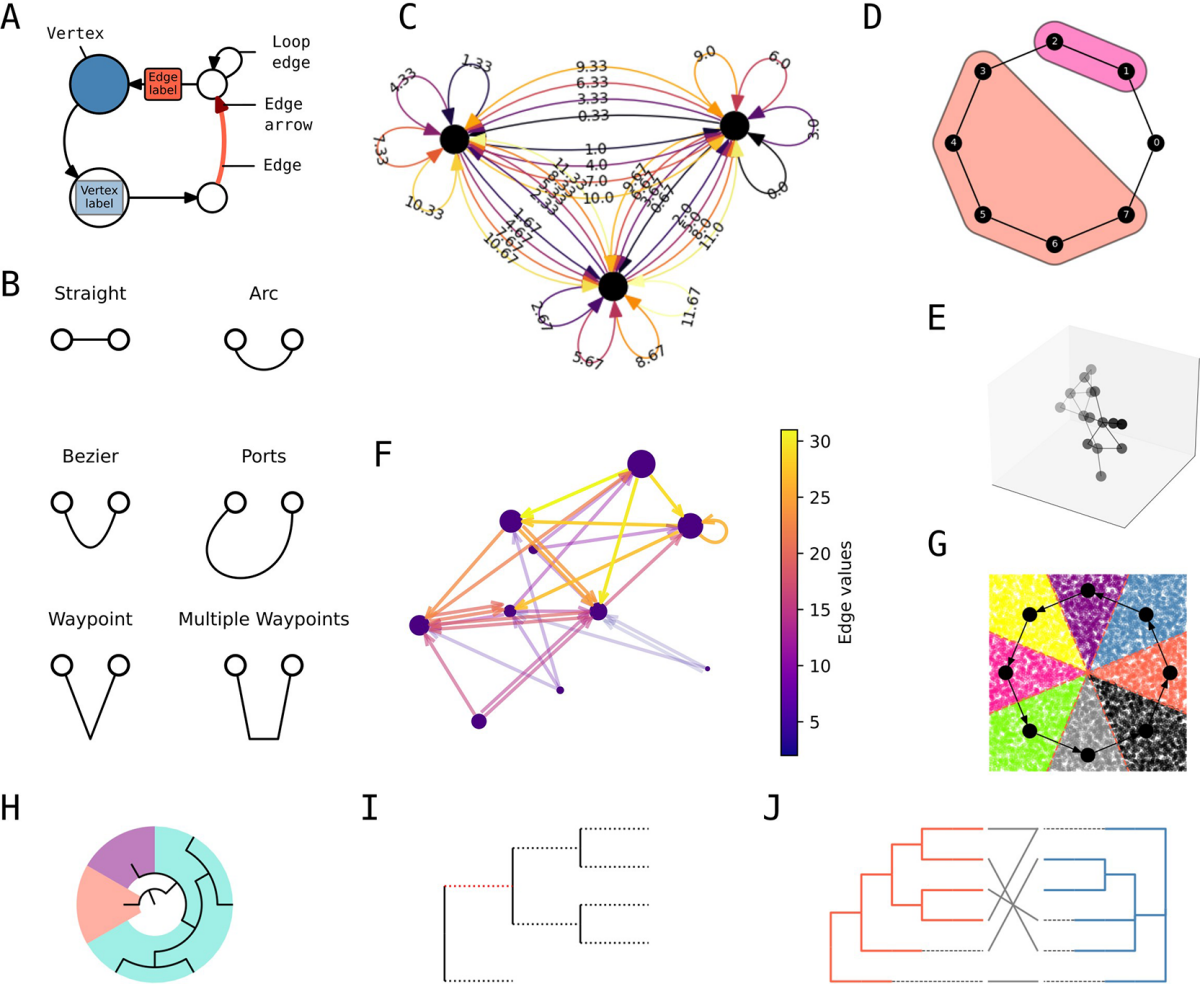
Style grammar and features. **A)** Style grammar for network visualisations. **B)** Edge geometry grammar. **C)** Example of global (e.g. vertex colour) and per-element styling (e.g. edge colour and curvature). **D)** Vertex clustering visualisation. **E)** Three dimensional visualisation with depth shading. **F)** Quantitative edge colouring with colour bar. **G)** Combination of network plot with *scipy*’s Voronoi tassellation [REF]. **H)** Cascading patches on a radial tree. **I)** Split edge styling on a rectangular tree. **J)** Dual tree visualisation with links between the leaves.

*iplotx* was designed around a declarative styling grammar that covers all elements types of the visualisation including vertices, edges, arrows, loops, and labels ( Figure 2A). The appearance of each element type can be customised in multiple ways. For example, edge geometry for a simple network with two nodes can follow a straight line, a circular arc, Bezier control points, specified incidence angles (called “ports” in *graphviz* ( Gansner and North 2000)), or waypoints ( Figure 2B). Furthermore, each element type can be specified either globally or on a per-element basis. For instance, one could set a global vertex colour and size but distinct colour and curvature for each edge ( Figure 2C). A library of default styles is maintained for rapid prototyping and can be used as a basis for further customisation. A complete style format specification that includes all possible style options is part of the public documentation.

*iplotx* also includes features that are either unique or found in distinct subsets of other packages. That includes - but is not limited to - graph clusterings and covers a la *igraph* ( Figure 2D), three-dimensional visualisations with node and edge depth shading ( Figure 2E), and quantitative element colouring via a colour map ( Figure 2F, similar to but easier to use than *NetworkX*’s functionality). Moreover, *iplotx* visualisations are compatible with other packages within the Matplotlib framework including seaborn for statistical data ( Waskom et al. 2014), pandas for tabular data ( McKinney 2011), scipy for scientific data ( Virtanen et al. 2020), and Cartopy for geolocation data ( Met Office 2010 - 2015). As an example, network visualisations can be combined with *scipy*’s Voronoi tassellation functionality ( Figure 2G). Tree visualisations also support dedicated features, among others: cascading patches for subtrees ( Figure 2H), split-edge styles ( Figure 2I), and a specialty function for pairs of trees facing each other, a common need in coevolutionary studies ( Figure 2J). For both networks and trees, *iplotx* also supports animations, post-render artist editing, and interaction via callback functions.

Crucially, by virtue of accepting many kinds of live objects as input ( Figure 1A), *iplotx* makes *all* features available to *all* packages.

## Use cases

**Figure 3.**
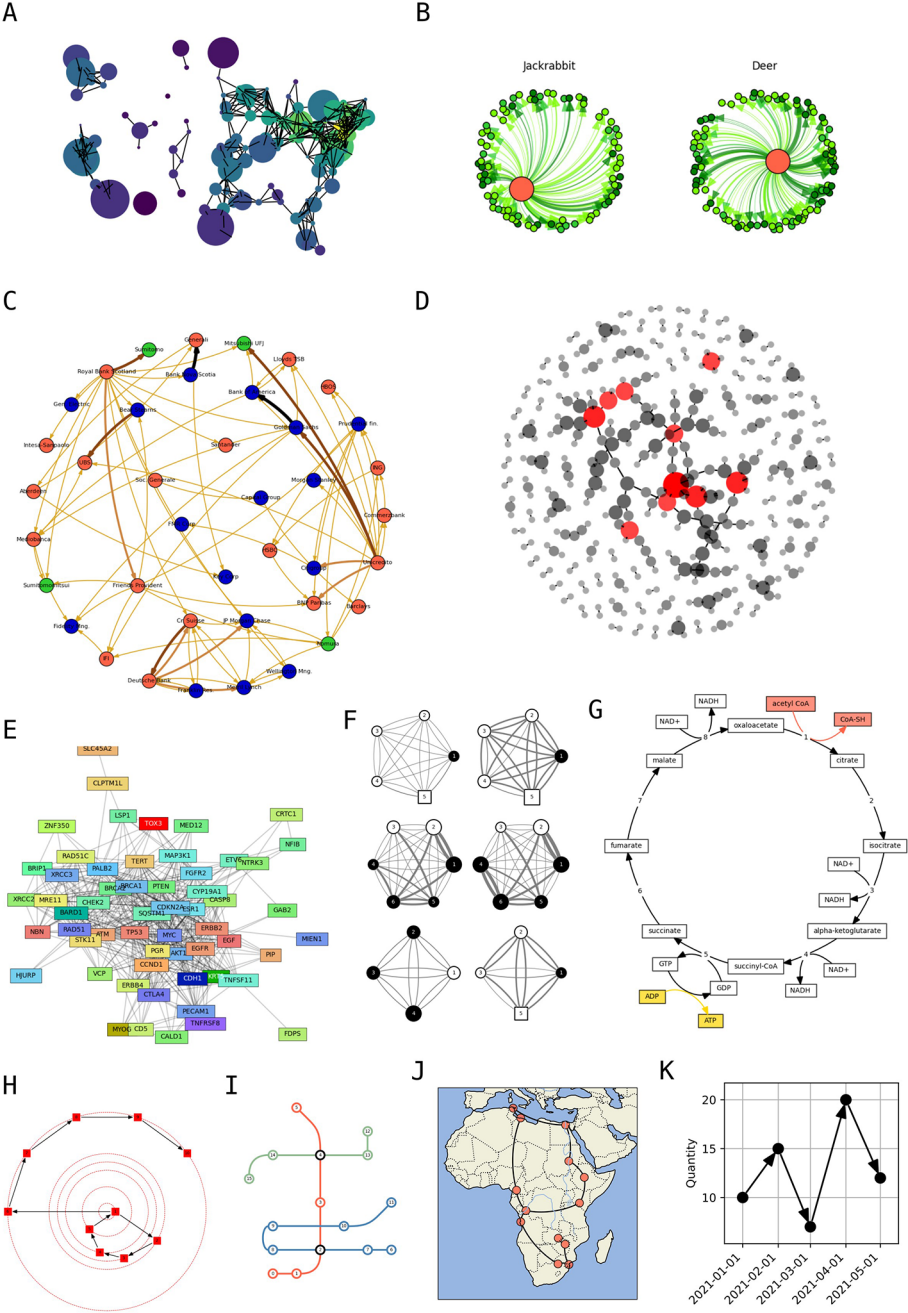
Applications across disciplines. **A)** Knuth’s network of distances between US cities, a classic example of network visualisation ( Knuth 1993). **B)** Molecular ecological network of food sources ( Meyer et al. 2020). **C)** Visualisation of a network of financial institutions ( Schweitzer et al. 2009). **D)** Protein-protein interaction network in *Escherichia coli* from the STRING database, with nodes (protein) coloured by their degree ( Szklarczyk et al. 2023). **E)** Protein-protein interaction network of breast cancer genes ( Szklarczyk et al. 2023). **F)** Foraging strategy models in a cooperatively breeding primate (De la Fuente et al. 2022). **G)** Central part of the citric acid cycle that underpins cellular respiration ( Kanehisa and Goto 2000). **H)** Example of social network from *SocNetV* (“Free and Open-Source Tool for Social Network Analysis,” n.d.). **I)** Example of a transportation network diagram with three train lines. **J)** Network of African cities onto a Cartopy geographic projection ( Met Office 2010 - 2015). **K)** Example of an *iplotx* network visualisation interpreted as a quantitative data chart.

Given that networks are widely used across scientific disciplines, a large gallery of examples specific for individual fields was created. In addition to classic examples from network science such as Donald Knuth’s map of US cities ( Figure 3A) ( Knuth 1993), it includes molecular ecological networks ( Figure 3B) ( Meyer et al. 2020), economic networks between financial institutions ( Figure 3C) ( Schweitzer et al. 2009), protein-protein interaction networks within bacteria ( Figure 3D) and in cancer ( Figure 3E) ( Szklarczyk et al. 2023), models for foraging networks in primates ( Figure 3F) (De la Fuente et al. 2022), diagrams for biochemical reaction chains ( Figure 3G) ( Kanehisa and Goto 2000), social network tutorials from *SocNetV* ( Figure 3H) (“Free and Open-Source Tool for Social Network Analysis,” n.d.), conceptual charts for train lines and stations ( Figure 3I), and geographic maps with non-Cartesian geometries ( Figure 3J) ( Met Office 2010 - 2015). Moreover, because of its tight integration with Matplotlib, *iplotx*’s visualisation can be used to encode generic quantitative data, extending all styling options to any chart type ( Figure 3K).

## Discussion

The landscape of network and tree analysis software tools is rich and diverse, catering to different types of users that require speed ( Antonov et al. 2023), a simple programmatic interface ( Hagberg, Schult, and Swart 2008), or complex GUIs ( Shannon et al. 2003). This diversity, however, also causes feature and format fragmentation. *iplotx* is a direct attempt to counter this phenomenon and thereby equip many existing network and tree analysis packages with a powerful visualisation toolkit.

While this package focuses on Python, *ggraph* follows similar principles in R ( Pedersen 2025), in particular the consideration of analysis, layout, and visualisation of networks as distinct tasks. Compared to *ggraph*, *iplotx* includes some additional features such as three-dimensional visualisations, internal styles, a library of arrow and vertex shapes, and dedicated tree features such as cascades and layouts. *Ggtree* is an R library that specialises in tree layout and visualisation ( Yu et al. 2017) and does not support general networks. Unlike *ggtree*, *iplotx* does not include any functions to directly read trees or network statically stored in files or the web. This is a design choice as many of the tree analysis libraries include parsers for many file formats already ( Huttley et al. 2025; Huerta-Cepas, Serra, and Bork 2016; Cock et al. 2009; Team 2020; Moreno, Holder, and Sukumaran 2024) and there is no reason for *iplotx* to duplicate this functionality.

The central tenet of *iplotx*, to democratise rich customisation options to *all* input packages, also represents a form of architectural robustness. Whether or not each individual analysis package is maintained in the future, users will be able to build network visualisations with the exact same appearance via another package. At the same time, reliance on a high-level rendering backend - Matplotlib ( Hunter 2007) - greatly expands the compatibility of the visualisations with dozens of other packages that use the same framework (e.g. ( Waskom et al. 2014; Virtanen et al. 2020)). This design choice, which echoes *ggraph* ( Pedersen 2025) but differs from other packages ( Huerta-Cepas, Serra, and Bork 2016; Peixoto 2017), facilitates onboarding for a majority of users unfamiliar with low-lever renderers such as Cairo and GTK.

In conclusion, *iplotx* is a universal network and tree visualisation framework that democratises a large set of styling options to all network analysis packages in Python.

## Software availability statement

Source code available from: https://github.com/fabilab/iplotx.

Archived software available from: https://doi.org/10.5281/zenodo.17585402.

( Fabio Zanini, & Matthew Andres Moreno. (2025). fabilab/iplotx: v1.5.1 (1.5.1). Zenodo.)

License: MIT.
