# Supplementary material for: A universal tool for visualisation of networks and trees in Python
Source: F1000Res. 2025 Dec 9;14:1377. [Version 1] doi: 10.12688/f1000research.173131.1 (PMC12946695; doi:10.12688/f1000research.173131.1)
Supplement: Peer review history

# Reviewer report: Reviewer response for version 1

**DOI:** 10.5256/f1000research.190914.r445964

**Published:** 2026-02-26

**Authors:** Valeria Velásquez-Zapata

**Affiliations:** 1 GreenLight Biosciences, Durham, NC, USA

This article presents iplotx, a universal Python package designed to provide visualization of networks and trees compatible with several libraries. The rationale for the software are clearly explained, addressing a real need for interoperability and customizability that existing tools lack. The paper thoroughly documents the design choices, integrations, and support for a broad range of input formats and outputs. Performance benchmarks and extensive examples across diverse scientific domains. The author shows that iplotx can deliver identical visualizations regardless of input source. The demonstration of scalability and cross-discipline applicability reinforce the claim that iplotx is a robust and broadly useful solution. When checking for software sources and documentation, I could verify the resources offer a aseries of examples for network visualization and on how to install and use the tool. Overall, the article is well written and the software a valuable addition to the Python ecosystem for network visualization.

Are the conclusions about the tool and its performance adequately supported by the findings presented in the article?

Yes

Is the rationale for developing the new software tool clearly explained?

Yes

Is the description of the software tool technically sound?

Yes

Are sufficient details of the code, methods and analysis (if applicable) provided to allow replication of the software development and its use by others?

Yes

Is sufficient information provided to allow interpretation of the expected output datasets and any results generated using the tool?

Yes

Reviewer Expertise:

BIoinformatics

I confirm that I have read this submission and believe that I have an appropriate level of expertise to confirm that it is of an acceptable scientific standard.

# Reviewer report: Reviewer response for version 1

**DOI:** 10.5256/f1000research.190914.r451458

**Published:** 2026-02-03

**Authors:** Adam L Tyson

**Affiliations:** 1 University College London, London, UK

This paper outlines *iplotx, *a general purpose network plotting library. This software presents a unified interface for plotting networks generated by different open-source tools. This is a simple tool, but it fulfils a real need, and does it well. The paper is nicely written, and the software itself follows all software development best practices, with linting, testing, type hints, docstrings and continuous integration set up etc. The software is freely available on PyPI and is easy to install (at least on this reviewers machine). The documentation is particularly good, with reference API documentation, details on how to customise the visualisation, along with an extensive gallery of examples.

 There are limited details about the code and methods within the paper, however, there is plenty of detail within the online documentation and within the code itself (where it should be!). 

 There are a few minor issues within the paper:

 1. It is stated that iplotx “guarantees” that networks will be plotted identically regardless of the source software. “Guarantee” is a strong term, I would either provide evidence that this is always true, or just soften the language - maybe “ensures”?

 2. Fig 1 and Fig 2 seem to be missing references in the caption.

 3. In the results section, it should read “covers all element types of the visualisation”, i.e. make “elements” singular.

 4. Only part of “De la Fuente et al. 2022” links to the reference. 

 Other than these, the tool and the paper are well written, and seem to address a real need in an efficient and well-thought out manner.

Are the conclusions about the tool and its performance adequately supported by the findings presented in the article?

Yes

Is the rationale for developing the new software tool clearly explained?

Yes

Is the description of the software tool technically sound?

Yes

Are sufficient details of the code, methods and analysis (if applicable) provided to allow replication of the software development and its use by others?

Yes

Is sufficient information provided to allow interpretation of the expected output datasets and any results generated using the tool?

Yes

Reviewer Expertise:

Software engineering, Python, neuroscience, image analysis.

I confirm that I have read this submission and believe that I have an appropriate level of expertise to confirm that it is of an acceptable scientific standard.
